# Rapid improvement in verbal fluency and aphasia following perispinal etanercept in Alzheimer's disease

**DOI:** 10.1186/1471-2377-8-27

**Published:** 2008-07-21

**Authors:** Edward L Tobinick, Hyman Gross

**Affiliations:** 1 Institute for Neurological Research, a private medical group, inc., Los Angeles, USA; 2 Department of Neurology, USC School of Medicine, Los Angeles, USA

## Abstract

**Background:**

Recent clinical studies point to rapid and sustained clinical, cognitive, and behavioral improvement in both Alzheimer's disease and primary progressive aphasia following weekly perispinal administration of etanercept, a TNF-alpha inhibitor that acts by blocking the binding of this cytokine to its receptors. This outcome is concordant with recent basic science studies suggesting that TNF-alpha functions *in vivo *as a gliotransmitter that regulates synaptic function in the brain. We hypothesized that perispinal etanercept had the potential to improve verbal function in Alzheimer's disease, so we included several standarized measures of verbal ability to evaluate language skills in a clinical trial of perispinal etanercept for Alzheimer's disease.

**Methods:**

This was a prospective, single-center, open-label, pilot study, in which 12 patients with mild-to-severe Alzheimer's disease were administered etanercept, 25–50 mg, weekly by perispinal administration for six months. Two additional case studies are presented.

**Results:**

Two-tailed, paired t-tests were conducted comparing baseline performance to 6-month performance on all neuropsychological measures. Test batteries included the California Verbal Learning Test-Second Edition, Adult Version; Logical Memory I and II(WMS-LM-II) from the Wechsler Memory Scale-Abbreviated; the Comprehensive Trail Making Test (TMT); Boston Naming Test; and letter(FAS) and category verbal fluency. All measures revealed a significant effect except for the Boston Naming Test and the TMT-4, with WMS-LM-II being marginally significant at p = .05. The FAS test for letter fluency was most highly significant with a p < 0.0007. In addition, rapid improvement in verbal fluency and aphasia in two patients with dementia, beginning minutes after perispinal etanercept administration, is documented.

**Conclusion:**

In combination with the previously reported results of perispinal etanercept in Alzheimer's disease and primary progressive aphasia, these results further argue that larger scale studies of this therapeutic intervention, including Phase 3 trials, are warranted in dementias. In addition, these results may provide insight into the basic pathophysiologic mechanisms underlying Alzheimer's disease and related forms of dementia, and suggest the existence of novel, rapidly reversible, TNF-mediated pathophysiologic mechanisms in Alzheimer's disease which are worthy of further investigation.

## Background

Substantial and increasing clinical, genetic, epidemiologic, and basic science evidence supports a central role of excess tumor necrosis factor-alpha (TNF-alpha) in the pathogenesis of Alzheimer's disease, suggesting that excess TNF-alpha is a therapeutic target [1-19].

Etanercept, a recombinant dimeric fusion protein consisting of the extracellular ligand-binding portions of two human p75 TNF-alpha receptors linked to the Fc fragment of human IgG1, binds to TNF-alpha and blocks its interaction with cell surface TNF-alpha receptors, thereby reducing the biologic effect of excess TNF-alpha [20]. The increasing evidence supporting a central role of TNF-alpha in Alzheimer's suggested that, if appropriately administered, etanercept, already FDA-approved for certain inflammatory conditions mediated by TNF-alpha, such as rheumatoid arthritis, might be efficacious in Alzheimer's. In addition, in contrast to anti-TNF monoclonal antibodies, such as infliximab, etanercept also binds to and suppresses the action of lymphotoxin (formerly known as TNF-beta), the physiologic significance of which in Alzheimer's is not presently known [21,22].

Perispinal administration of etanercept had been previously reported to be rapidly effective(within minutes) in providing relief of intractable pain associated with lumbar and cervical radiculopathy [23-26]. These findings, which were consistent with the idea that perispinal administration enabled etanercept to cross the blood-dural barrier, led to the expanded concept of the potential of the bi-directional cerebrospinal venous system as a route of delivery of therapeutic molecules to both the spine and the brain [1-3,23-27]. Specifically, it was conceived that etanercept, and potentially other large molecules, could be delivered to the brain by perispinal administration and subsequent retrograde carriage to the brain via the cerebrospinal venous system [1-3,25,27].

In 2006, the authors and their colleagues published an IRB-approved six month pilot study involving a cohort of 15 patients, that provided proof-of-concept that perispinal delivery of etanercept was effective for the treatment of Alzheimer's disease [2]. Clinical experience suggesting continued clinical effectiveness with maintenance treatment, continuing for more than two years in some patients, was reported in 2007 [1]. Most recently, rapid clinical, cognitive, and behavioral improvement, beginning within minutes of administration of perispinal etanercept, was documented in a patient with moderate dementia fulfilling the criteria for probable Alzheimer's [3].

Improved verbal abilities following perispinal etanercept was reported in some of the above studies [1-3]. This paper provides additional clinical data relevant to these reports, in patients with Alzheimer's disease, and in a related form of dementia in which patients present with prominent effects on verbal function, semantic dementia. An article by one of the authors documenting rapid improvement following perispinal etanercept in another form of dementia with prominent language dysfunction, primary progressive aphasia, has just published [28].

Semantic dementia, discussed in the first case report included, is considered by many to be a variant of frontotemporal dementia [29]. As with primary progressive dementia, semantic dementia is a progressive neurodegenerative disorder for which there is no established treatment. Excess TNF-alpha in the cerebrospinal fluid has been documented not only in Alzheimer's disease, but also in frontotemporal dementia and vascular dementia, so that all of these may be included in the category of TNF-alpha-mediated dementias [30,31].

### Clinical data

The clinical data included in this article is separated into two sections. In the first section, new data related to verbal function from an IRB-approved Phase 2 clinical research study involving a 15 patient cohort with Alzheimer's disease ranging in severity from mild to severe is reported. This data supplements a previous report of improvement in standard cognitive measures in this same 15 patient cohort which has been previously published [2]. The full methodology of this clinical trial utilizing perispinal etanercept has been previously reported [2].

In the second section case reports from two additional patients are included. These patients were not research subjects, but rather were treated with perispinal etanercept off-label as part of our usual practice of medicine.

### Clinical trial data

#### Methods

This IRB-approved clinical trial was performed prior to, and independent of, the two case reports described below. The methodology of this clinical trial has been previously reported [2].

The patient and the patient's son gave full consent to be identified in the video which accompanies this article and for the video's publication.

Briefly, the author and his colleagues designed and carried out an open-label, prospective, single-center, open-label, pilot (proof-of-concept) study of 15 patients with probable Alzheimer's disease ranging in severity from mild to severe treated weekly with perispinal etanercept for a period of six months [2]. This clinical trial began in 2004 after institutional review board approval was obtained [2]. The study was also registered in the clinical trial database maintained by the National Institute of Health (NCT00203359). Improvement in the main outcome measures, which were the Mini-Mental State Examination (MMSE) [32], the Alzheimer's Disease Assessment Scale-Cognitive subscale (ADAS-Cog) [33], and the Severe Impairment Battery [34], has been previously reported [2].

During the 15 patient pilot study, in addition to the standard cognitive measures above, additional neuropsychological test batteries were administered, many of which carefully measure verbal function. The test batteries used included the California Verbal Learning Test-Second Edition, Adult Version (CVLT-II) [35], Logical Memory I (LMI), and II (LMII) from the Wechsler Memory Scale-Abbreviated (WMS-a) [36], the Comprehensive Trail Making Test (TMT) [37,38], Boston Naming Test [39], and letter(FAS) and category verbal fluency [40-42].

Neurocognitive assessments were conducted monthly. Three of the 15 patients in the pilot study with severe dementia were excluded as they could not be reliably assessed with these tools.

#### Results

**Figure 1:**
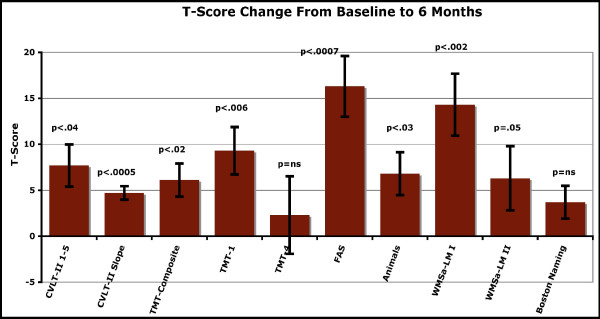
**Changes in verbal fluency, learning, and memory over 6 months during maintenance treatment with perispinal etanercept in a 12 subject pilot study, as reflected by T-score change from baseline to 6 months.** Two-tailed, paired t-tests were conducted comparing baseline performance to 6-month performance on all neuropsychological measures. Abbreviations: California Verbal Learning Test-Second Edition, Adult Version (CVLT-II), including tests 1–5 and slope of change; Logical Memory I (LMI), and II (LMII) from the Wechsler Memory Scale-Abbreviated (WMS-a); the Comprehensive Trail Making Test (TMT), including tests 1 (TMT-1) and 4 (more difficult) (TMT-4); Boston Naming Test; and letter(FAS) and category(animal) verbal fluency.

Two-tailed, paired t-tests were conducted comparing baseline performance to 6-month performance on all neuropsychological measures. T-tests were conducted comparing baseline assessment scores to 6-month assessment scores. Standard T scores were used in the CVLT-II 1–5, TMT, and Verbal Fluency analyses, and standard z-scores were used for WMSa, and secondary CVLT-II measures analyses, while raw values were used for the Boston Naming Test. As can be seen from the figure (Figure 1), all measures revealed a significant effect except for the Boston Naming Test and the TMT-4, with WMS-LM-II being marginally significant at p = .05. The FAS test for letter fluency [40-42] was most highly significant with a p < 0.0007.

## Case Reports

### Case report one, Progressive Aphasia, Semantic Dementia

The patient is a 78 y.o. man with a six year history of progressive dementia beginning with forgetfulness, followed by progressive paucity of speech and associated language difficulties. Three years before he began speaking with predominantly one word answers to questions. In July 2005 he had a neuropsychological evaluation which demonstrated a variety of language difficulties, with paraphasic errors with word substitutions and mispronunciations. There were obvious impairments in attention, linguistic processing, and memory. He had dysnomia, oral fluency problems, and articulation difficulties. In September 2005, it was noted that he began using word substitutions, such as "hotto" for "honda", "tea" for "tie", and "burgle" for "verbal". There were no changes in mood, personality, or behavior, and no depression, hallucinations, or delusions. In April 2006, he was evaluated by a local neurologist and found to have severe aphasia. Work-up included an MRI, which showed global volume loss and minimal small vessel occlusive disease. CBC, RPR, B12, folate and TSH were normal. A diagnosis of possible primary progressive aphasia was made. Memantine was begun. A second opinion from another neurologist was obtained on April 25, 2006. His MMSE was 12/30. There was severe aphasia and word-finding difficulty and severely impaired comprehension, naming and word repetition. A diagnosis of semantic dementia, a language variant of frontotemporal dementia [29,43], was made. Memantine was increased to 10 mg twice a day, but caused constipation and was discontinued. In May 2006, donepezil was begun at 5 mg per day and increased to 10 mg daily the following month. By September 2006, the patient's memory difficulties had progressed such that he could not remember events from the same day. His speech was limited to only a few single words. Memantine was re-started. By February 2007, he was having difficulties performing the normal activities of daily living. He required assistance with showering, and on examination was almost mute. By May 2007, his comprehension of spoken commands had worsened, accompanied by marked apathy. Memantine was discontinued because of excessive daytime somnolence.

On February 6, 2008, the patient was brought to our clinic for evaluation. He had been mostly mute for more than four months. There was no history of head trauma, tremor, or significant active medical illness. The family specifically denied a history of demyelinating disease, congestive heart failure, bleeding disorder, diabetes mellitus, lymphoma, blood disorder, hepatitis, immunosuppression, or exposure to tuberculosis. There was a previous history of disc surgery in 1997. Present medications were donepezil 5 mg per day and memantine 10 mg twice a day. The patient's neurological examination was unremarkable except for the mental status exam. The patient had no spontaneous speech, did not verbally respond to most requests, and was unable to follow any but the simplest commands. Laboratory examination was unremarkable, with a normal CBC, serum folate, free T4 index, serum vitamin B12, creatinine, and hemoglobin A1c. PPD skin testing for tuberculosis was negative.

After written informed consent was obtained, as part of our usual practice of medicine, perispinal etanercept (25 mg) was administered by injection to the posterior neck in the midline followed by Trendelenburg positioning, as previously described [2]. Administration was tolerated without difficulty. Ten minutes after injection the patient was able to repeat single numbers, and was asked if he felt better. He replied, "I don't know". The patient's son, who spoke about his father's improvement that day and eight days later (Additional file 1), commented that the improved speech output following perispinal etanercept was highly significant. The patient returned to our clinic for follow-up on February 14 at which time his son reported that his father was better in many ways. His behavior was improved as was his ability to follow spoken commands; his verbal output of intelligible words was notably increased, although the words were few and usually single; and he was walking faster than he had been. At one month the patient returned with his son who reported that all of his previously reported clinical improvement had been sustained for the entire month.

### Case report two, non-fluent aphasia secondary to Alzheimer's disease

This 80 y.o. married, right-handed engineer presented to our clinic with a two and one-half year history of increasing word-finding difficulty, which had become much more prominent in the past six months. This was accompanied by a less prominent disturbance of short-term memory during the past six to twelve months. Also during the past six months the wife reported gradual deterioration in his handwriting and increasing difficulty in handwriting notes. He continued driving to work. He remained independent and able to perform all of the normal activities of daily living. No changes in personality were noted. There was no history of hallucinations, motor symptoms, seizures, or apathy.

Past medical history included pericarditis with possible viral myocarditis, atrial fibrillation, benign prostatic hypertrophy, hypothyroidism, and increased cholesterol. There was no history of demyelinating disease, substance abuse, congestive heart failure, bleeding disorder, diabetes mellitus, lymphoma, blood disorder, hepatitis, immunosuppression, or exposure to tuberculosis. Three years before the patient had an automobile accident with a brief loss of consciousness. Current medications were tamsulosin, dutasteride, diltiazem, donepezil, levothyroxine, atorvastatin, and warfarin.

**Figure 2:**
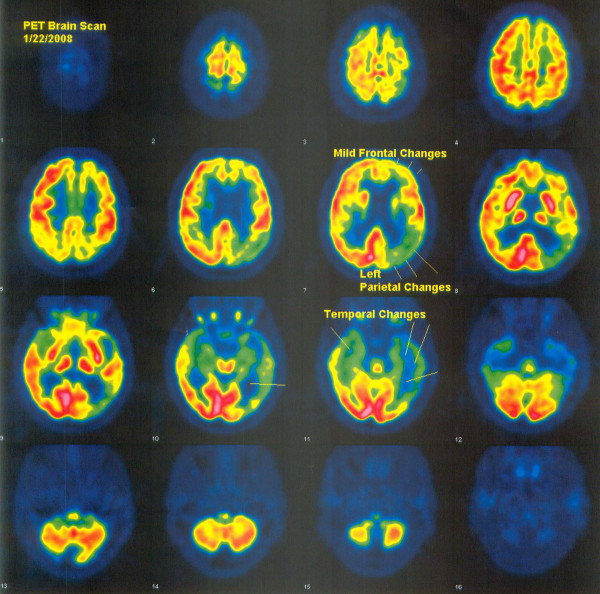
FDG-PET brain scan of a patient with non-fluent aphasia secondary to Alzheimer's disease (case report 2), prior to treatment, demonstrating decreased glucose metabolism in the left parietal, bilateral temporal, and left frontal lobe.

Laboratory testing was unremarkable, with normal white blood cell count, hemoglobin 13.4, hematocrit 39.5, platelets 275,000, INR 1.0, and normal serum chemistries, BUN, serum vitamin B12 and folate, RPR, TSH and T4, and chest x-ray. PPD skin testing for tuberculosis was negative. Carotid duplex study on November 22, 2006 showed plaque formation in the bilateral carotid bifurcation without any hemodynamically significant stenosis. MRI of the brain had previously documented moderate atrophy and nonspecific periventricular white matter changes consistent with microvascular white matter ischemic changes. FDG-PET of the brain on January 22, 2008 showed hypometabolism out of proportion to the degree of atrophy in the left parietal and left temporal regions (Figure 2). There was also hypometabolism of a lesser degree including the left cingulate cortex and left occipital region (Figure 2). These findings were read by the neuroradiologist as most consistent with a primary neurodegenerative process, probably asymmetric Alzheimer's disease.

On examination, the patient's blood pressure was 135/77, he was afebrile, with pulse 60 and irregularly irregular, respiratory rate 12, and lungs clear to auscultation. He was pleasant and cooperative, with obvious word-finding difficulty even in casual speech. His spontaneous and primary language was English. There was a prolonged latency of verbal response to most questions, with obvious circumlocution with pronouns for noun substitution associated with mild anxiety. Affect seemed slightly flat.

He was oriented to the year and month but not the calendar date, day of the week, city or location. On fund of knowledge testing, he could name the present and prior president of the U.S. but none past Clinton.

He had obvious impairment in naming common objects; specifically he could not name a comb, coin, toothbrush or key. He could not identify pictures of a camel or rhinoceros but could identify a lion. On the first 10 items of the short form of the Boston Naming Test [39] he could only identify correctly a house and bench; he used marked circumlocutions for the remainder of the items. He was able to accurately state his birthplace, place of birth and age and number of minutes in an hour but not the number of days in a year. On a phonemic fluency test (FAS) [40-42] he could name only three words beginning with the letter S in 60 seconds prior to perispinal etanercept treatment. On a semantic task for category fluency he could list only three animals over 30 seconds.

He had marked difficulty with memory and took five repetitions to register five memoranda, but could not retrieve any of these memoranda after 90 seconds, despite categorical cueing and multiple choice cueing. When asked to recite the days of the week in reverse order starting with Sunday he could only recite Sunday and Saturday.

Cranial nerves II through XII were intact. He had a normal fundoscopic examination and visual fields were full to confrontation.

Deep tendon reflexes were symmetrical, with supinator and biceps two plus, triceps one plus, quadriceps one plus, and absent ankle reflexes. Toe signs were flexor. There was full strength throughout with normal station and gait. There was no atrophy, fasciculations, or grasping. There was a positive palmomental reflex bilaterally. There was a mild intention tremor, but no resting tremor, rigidity, ataxia, or dystonia. Sensation was intact to pinprick and light touch throughout.

The patient had difficulty with mathematical calculations. Prior to treatment he could not divide 58 by two and could not add 29 plus 11. MMSE score was 18/30. Montreal Cognitive Assessment score was 12/30 [44]. His Cognitive Abilities Screening Instrument (CASI) [45] score was 69.2, all consistent with moderate cortical dementia.

After written informed consent was obtained, he was treated, as part of our usual practice of medicine, with perispinal etanercept (25 mg) administered to the posterior neck in the midline followed by Trendelenburg positioning. This was tolerated without difficulty.

Eight minutes after perispinal etanercept the patient was re-examined. He seemed more alert. His speech seemed less effortful. He could recite all seven days of the week backwards, which he could not do before the treatment, and correctly calculated 58 divided by two.

The patient and his wife flew home to New York. They returned three weeks later, at which time the patient received a second dose of perispinal etanercept 25 mg. The wife reported that during the weeks following the first dose, the patient was improved in his conversational ability, attention span, and forgetfulness. These improvements were particularly noticeable in the first week after treatment. No adverse effects of treatment were noted. On examination the patient's verbal fluency was notably improved, he spoke with greater ease, and with less latency of response to questions. In casual conversation he appeared to have less word-finding difficulty. Ten minutes after this second dose his speech appeared further improved.

The patient and his wife again flew back to New York and returned to California three weeks later. His wife reported continued improvement, but again noted greater improvement during the first week following the perispinal etanercept dose. She reported improvement in his ability to handwrite notes and conversational ability, as well as some improvement in his organizational skills. In addition, examination prior to his third dose of perispinal etanercept revealed improvement in affect and attention. His speech was clearly more fluent, and testing of his phonemic fluency (FAS) for the letter S was notable (11 words in 60 seconds) as it demonstrated a marked improvement from his performance prior to his first perispinal etanercept treatment six weeks before. Although with some difficulty, he was also able to recite the days of the week in reverse order, which he had not been able to do at all prior to treatment. Montreal Cognitive Assessment score was 14/30 [44]. A third dose of perispinal etanercept 25 mg was administered. Because the improvement was greatest during the week following each of the two previous treatments, the patient was advised to attempt weekly dosing.

## Discussion

The data from this six month Phase 2 clinical trial in 12 patients with Alzheimer's disease suggest notable improvements in verbal learning, memory, and fluency in this cohort treated with perispinal etanercept. This data is consistent with the preliminary evidence from the two case reports included herein, and with the case study most recently reported by the authors [3,4]. The data reported here, along with confirmatory clinical experience of more than three years duration in Alzheimer's disease, suggest that perispinal etanercept treatment of patients with TNF-alpha mediated dementias may result in improvement in verbal fluency and related language functions [1-4]. In addition, these data, combined with those from previous reports, suggest that positive clinical effects may begin rapidly, within minutes, and may be durable with ongoing maintenance dosing [1-4]. Perispinal etanercept may result in improvement in behavior, frontal lobe and executive function, conversational abilities, naming abilities, and ability to comprehend and follow spoken commands [1-4]. The clinical effects suggest that this treatment approach, in addition to its utility for other forms of Alzheimer's disease, may be useful for patients with frontotemporal dementia and frontal variant Alzheimer's disease [46]. Further study will be necessary to characterize response rates, dosing schedules, and duration of response in the non-Alzheimer dementias.

The family and clinician observations of improvements in the patients' cognition, verbal ability, and behavior, suggest the possibility that perispinal etanercept may have the ability to reduce the caregiver burden in selected patients with severe dementia. One may argue that these are observations in only a small number of patients. But rather than dismissing the potential scientific significance of these observations, it would be more scientifically appropriate to view these results as observations which merit further investigation [47]. Small studies and case reports lend themselves to examination of individual treatment responses, particularly with diseases which are well-studied and characterized [47]. In a study such as this, the first objective is often to establish the likelihood of a biological effect beyond the chance of a type I error, i.e., whether any individual experienced a significant treatment effect [47]. Further study will be necessary to characterize response rates, dosing schedules, and duration of response.

As previously hypothesized, the current results suggest that perispinal etanercept may have the ability to influence brain function, perhaps through delivery via the cerebrospinal venous system [1,3,25]. This form of delivery may be facilitated by the large surface area of the choroid plexus (which may be as much as one-half the size of the entire surface area of the cerebral capillaries) and by the decreased barrier characteristics of the choroid plexus [48,49]. Etanercept reaching the choroid plexus could have widespread neuronal effects due to its effect on glia, even in the absence of deeper brain delivery, due to the widespread effects exerted on the multiple synapses even a single glial cell can control, through their extensive projections [3,50-52].

As previously discussed, the rapid clinical improvements seen following perispinal etanercept may be due to synaptic effects related to the role of TNF-alpha as a gliotransmitter [3,50-52]. Other molecules which function as gliotransmitters are adenosine, glutamate, ATP, and D-serine [50-52]. Because of the related pro-inflammatory effects of TNF-alpha and IL-1, initially one might be tempted to speculate that IL-1 might have synaptic effects similar to those of TNF-alpha. Caution, however, may be in order, because these cytokines may have quite divergent clinical effects [53]. Indeed, it is quite likely that the effects of different cytokines may vary across the different dementias, and even within the spectrum of diseases which we currently classify together as different forms of Alzheimer's disease [54].

In addition to synaptic effects, etanercept may have vascular effects which may contribute to both the rapid and sustained clinical improvement noted. Etanercept may have the potential to improve endothelial dysfunction and thereby have a vasculoprotective role [8]. In addition, etanercept may have the potential to improve microvascular function, particularly in a clinical disorder associated with TNF excess [55]. This may be of particular importance in patients treated with perispinal etanercept who have a vascular component to their dementia, such as patients with mixed dementia consisting of Alzheimer's disease and vascular dementia.

Anatomically targeted delivery of etanercept, tailored to the disease target, may be critically important to ensure the success of the intervention [1,2,23-25,56-58]. Delivery of etanercept into an anatomic structure where it would not have access to the primary site of pathology, such as intradiscal administration for treatment of radiculopathy, may doom such an intervention to failure [57]. This highlights the concern of the authors that physicians inexperienced with perispinal administration of etanercept may attempt to initiate etanercept treatment in patients with dementia utilizing the routes normally used to treat rheumatoid arthritis and psoriasis patients i.e. subcutaneous administration in the abdomen, arms, or thighs. Published data do not support the concept that etanercept can reach the cerebrospinal fluid in therapeutic concentration if given by its usual method of subcutaneous administration in the abdomen [59,60]. Etanercept does not cross the blood-brain barrier when administered systemically [59]. Conversely, when delivered via perispinal administration into Batson's plexus, large molecules may have the ability to reach the brain via retrograde delivery through the cerebrospinal venous system, a potential anatomic route first demonstrated by Batson in cadavers [1,27,61].

In addition to concerns regarding drug delivery, dosage and dosing intervals may need to be individualized for each patient. This requires experience with the use of perispinal etanercept in the treatment of patients with dementia. Potential side effects of the use of perispinal etanercept for the treatment of dementia, an off-label use, include all of the risks inherent with the use of etanercept for its labeled indications, which may include rare instances of death, infection, decreased blood counts, congestive heart failure, lymphoma, demyelinating disease, and reactivation of tuberculosis [62]. PPD skin testing prior to initiation of etanercept treatment is mandatory, and a black box warning highlighting the risk of tuberculosis, sepsis, and severe infection has been added to the package insert [62].

There are limitations to the data presented. The clinical trial was open-label, and not controlled. The data is limited. These caveats notwithstanding, the scientific rationale for the further investigation of anti-TNF-alpha treatment of Alzheimer's disease is compelling, with supporting genetic, epidemiologic, clinical, and basic science evidence [1-19,30,50,52]. In addition, family members, independent neurologists, and other independent observers have confirmed the clinical, cognitive, and behavioral improvement noted [1-4]. Nevertheless there is, as yet, no double-blind, placebo-controlled data, the availability of which would further strengthen the reported results. These additional clinical trial results in this 12 patient cohort, together with these case study results, further support the initiation of larger scale studies of this therapeutic intervention, including Phase 3 trials. In addition, these results may provide insight into the basic pathophysiologic mechanisms underlying Alzheimer's disease and related forms of dementia, and suggest the existence of novel, rapidly reversible, TNF-mediated pathophysiologic mechanisms in both Alzheimer's disease and semantic dementia which are worthy of further investigation.

## Abbreviations

TNF-alpha: tumor necrosis factor-alpha; AD: Alzheimer's disease; HG: Hyman Gross, MD; ET: Edward Tobinick, MD; MRI: magnetic resonance imaging; PET: positron emission tomography; FDG: fluorodeoxyglucose; NINCDS-ADRDA: National Institute of Neurological Communicative Disorders and Stroke-Alzheimer's disease and Related Disorders Association; MOCA: Montreal Cognitive Assessment; BUN: blood urea nitrogen; RPR: rapid plasma reagin screening test for syphilis; PPD: Purified Protein Derivative skin test for previous tuberculosis exposure; The California Verbal Learning Test-Second Edition, Adult Version: CVLT-II; Wechsler Memory Scale-Abbreviated (WMS-a) Logical Memory I: LMI; Wechsler Memory Scale-Abbreviated (WMS-a) Logical Memory II: LMII; Comprehensive Trail Making Test: TMT.

## Competing interests

Author Edward Tobinick has multiple issued and pending patents, assigned to TACT IP LLC, which describe the parenteral and perispinal use of etanercept for the treatment of Alzheimer's disease and other neurological disorders, including, but not limited to, U.S. patents 6015557, 6177077, 6419934, 6419944, 6537549, 6982089, 7214658 and Australian patent 758523. He owns stock in Amgen, the manufacturer of etanercept. In addition, he has pending patents which describe the use of the cerebrospinal venous system and/or perispinal administration to deliver other therapeutic or diagnostic agents to the brain, eye, spinal cord, and other anatomic structures.

Author Hyman Gross has no competing interests

## Authors' contributions

ELT wrote the text of the present article, with the exception of part of one case report; takes full responsibility for the entire content of the article; and gives approval for the final submitted version of the article. HG was primarily responsible for the design of the neurocognitive measures used in the initial pilot study, was one of the co-authors of the initial pilot study, and performed the neurological evaluation and wrote part of the case report on one patient included in the case series. He gives approval for the final submitted version of the article.

## Pre-publication history

The pre-publication history for this paper can be accessed here:



## Supplementary Material

Additional file 1 Video 1 Click here for file
